# Modeling the Interruption of the Transmission of Soil-Transmitted Helminths by Repeated Mass Chemotherapy of School-Age Children

**DOI:** 10.1371/journal.pntd.0003323

**Published:** 2014-12-04

**Authors:** James Truscott, T. Déirdre Hollingsworth, Roy Anderson

**Affiliations:** 1 London Centre for Neglected Tropical Disease Research, Department of Infectious Disease Epidemiology, Faculty of Medicine, Imperial College London, St Marys Campus, Norfolk Place, London, United Kingdom; 2 Mathematics Institute, University of Warwick, Coventry, United Kingdom; 3 School of Life Sciences, University of Warwick, Coventry, United Kingdom; 4 Department of Clinical Sciences, Liverpool School of Tropical Medicine, Liverpool, United Kingdom; Queensland Institute for Medical Research, Australia

## Abstract

**Background:**

The control or elimination of neglected tropical diseases has recently become the focus of increased interest and funding from international agencies through the donation of drugs. Resources are becoming available for the treatment of soil-transmitted helminth (STH) infection through school-based deworming strategies. However, little research has been conducted to assess the impact of STH treatment that could be used to guide the design of efficient elimination programs.

**Methodology:**

We construct and analyse an age-structured model of STH population dynamics under regular treatment. We investigate the potential for elimination with finite rounds of treatment, and how this depends on the value of the basic reproductive number *R_0_* and treatment frequency.

**Principal findings:**

Analysis of the model indicates that its behaviour is determined by key parameter groupings describing the basic reproduction number and the fraction of it attributable to the treated group, the timescale of material in the environment and the frequency and efficacy of treatment. Mechanisms of sexual reproduction and persistence of infectious material in the environment are found to be much more important in the context of elimination than in the undisturbed baseline scenario. For a given rate of drug use, sexual reproduction dictates that less frequent, higher coverage treatment is more effective. For a given treatment coverage level, the lifespan of infectious material in the environment places a limit on the effectiveness of increased treatment frequency.

**Conclusions:**

Our work suggests that for models to capture the dynamics of parasite burdens in populations under regular treatment as elimination is approached, they need to include the effects of sexual reproduction among parasites and the dynamics infectious material in the reservoir. The interaction of these two mechanisms has a strong effect on optimum treatment strategies, both in terms of how frequently to treat and for how long.

## Author Summary

The control or elimination of soil-transmitted helminth diseases through chemotherapy has recently become the focus of increased interest and funding from international agencies, charities, and pharmaceutical companies via drug donations for treatment in the poorer regions of the world. The design of treatment regimes and the interpretation of their impact benefit from analysis using robust and reliable mathematical models. By analyzing models of the effect of treatment on host parasite burden, we identify several aspects of parasite natural history and transmission which are often overlooked, but have a marked effect on the impact of treatment strategies. In particular, the inclusion of sexual reproduction and the dynamics of eggs or larval stages in the model changes the response of the parasite population to treatment when parasite burdens are low. This in turn has implications for the design of treatment strategies to eliminate parasites in terms of minimizing total drug use and the length of the program delivering them.

## Introduction

The neglected tropical diseases (NTDs) have been an increasing focus of attention in recent years as possible targets for infectious disease elimination or greatly improved control [1]. Donations from government aid agencies (e.g., U.S. Agency for International Development and Department for International Development), philanthropic organizations (Bill and Melinda Gates Foundation) and pharmaceutical companies have spurred these efforts, under the imprimatur of the World Health Organization [2]. One of the most prevalent infectious diseases under the NTD label is the group of intestinal nematodes referred to as the soil-transmitted helminths (STH) [3]. The three major parasite types in the broad classification of STH are *Ascaris lumbricoides* (roundworm), *Trichuris trichiura* (whipworm), and two species of hookworm (*Necator americanus* and *Ancylostoma duodenale*). Those infected can be treated by cheap and effective drugs with moderate to high efficacy for *A. lumbricoides* and hookworm but lower efficacy for T. trichiura [4], [5]. In the poorer regions of the world where these infections are prevalent, mass chemotherapy is typically employed to reduce the community wide burden of disease induced by these infections. Mass chemotherapy is frequently targeted at pre-school (pre-SAC: 2 to 4 years) and school-age (SAC) children (5 to 14 years of age), since this section of the population often harbor the highest burdens, are most at risk from developmental and social impact from their infestation and are most accessible to intervention (through school-based deworming programs).

Since these parasites do not induce protective immunity post expulsion by chemotherapy, an individual is re-infected and will often reacquire similar burdens of parasites to those that occurred prior to treatment [6]. As such, treatment must be repeated at intervals to maintain having a lasting effect. To date, rather little attention has been directed toward analyses of how best to design periodic chemotherapeutic interventions, in terms of who to treat, at what level of coverage and how often to get the biggest impact on parasite transmission and concomitant disease burden or in achieving elimination.

Mathematical models can be used to investigate the impact of different interventions on the evolution of the worm burden of the host population. Mathematical models of STH dynamics were first developed in the 1970s and 1980s and these models form the foundation of most subsequent work [7]–[9]. Many of the models developed more recently focus on how the distribution of worms in the host population is generated by the mechanisms of worm acquisition and loss by the host [10]–[14]. However, these models do not include the complete life-cycle of the parasite, and hence cannot address the treatment processes that interrupt the cycle. Several models have been developed that can describe the long-term development of the host worm burden, but these contain simplifying assumptions which we will show lead to significant biased behavior in the presence of regular treatment [8], [15], [16]. The model we present in this paper is a simplification of a fully age-structured model [9], [17]. It is similar to that employed by Chan et al. [15], but explicitly includes the dynamics of infectious material in the environment and sexual reproduction. Our overall aim is to use the insights derived from age-structured hybrid (deterministic and stochastic components) to refine the design of mass drug administration programs (MDA).

Analysis of the model reveals a set of key parameter groupings which control the model's response to regular chemotherapeutic treatment of different age groupings in the population. The key parameter groupings give insight into the most important mechanisms or groups of mechanisms for understanding the impact of treatment, and hence where efforts can best be directed in field studies to better parameterize intervention models. Particularly interesting is the interaction of sexual reproduction dynamics with the frequency and level of coverage of chemotherapeutic mass treatment. The insights derived are particularly relevant for scenarios in which elimination is the goal of MDA.

## Methods

A simple way to mimic non-random contact is to stratify the population into two age groups, namely; school-age children (5–14 years), and the rest (≤4 and ≥15 years). In reality, the younger section of this second age group probably plays a minimal role in the transmission process. The very youngest children will likely harbor relatively few parasites and will also not be engaged in community level transmission, being largely confined to the home. There is interest in treating the pre-SAC age group and other more sophisticated modeling work has addressed the impact, but within the current simple model, pre-SAC could be seen as an extension of SAC [18], [19]. As a result, we consider the rest of the population identified above essentially to be adults. Such a stratification of hosts groups has the further advantage of mimicking school-based treatment programs which are the most widely used vehicle for mass STH treatment. We assume that the child and adult age groups have negative-binomially distributed worm distributions with the same aggregation parameter, *k*, but different means, *M_c_* and *M_a_*, respectively. The means change over time independently according to the degree of contact of each group with a common infectious ‘reservoir’. The model equations are:
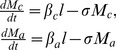
(1)


The quantity 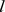 is the per capita infectiousness of the shared reservoir and *σ* is the inverse of the mean worm lifespan. The parameters 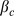 and 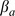 determine the strength of infectious contact with the reservoir for children and adults respectively. The absolute magnitude of these parameters is absorbed into *R_0_*, but their relative size is the chief determinant of the relative worm burdens in children and adults. Hence, by default, we set 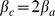, to approximately match the age profile found for *A. lumbricoides* [17]. The dynamics of the infectious reservoir are described by the following equation:
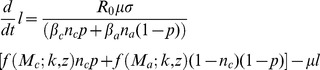
(2)


The quantities *p* and 1-*p* are the relative contributions of infectious material per capita for children and adults, respectively and the parameters *n_c_* and *n_a_* represent the proportion of the population in each age class. The parameter *μ* is the rate of decay of infectious material in the environment. The model described here differs from many of those previously developed [15], [16] by explicitly including the dynamics of the infectious reservoir. Assuming that infectious contact and contribution are aspects of the same process, we set 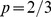. The function *f(M;k,z)* describes the mean egg production rate from a host population with mean worm burden *M*, distributed among the population with a negative binomial distribution (aggregation parameter *k*). It has the form
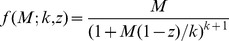
(3)


The parameter *k* is the shape parameter for distribution of worms among hosts, which is found to be approximately negative binomial across a range of species, and *z* describes the density-dependency of egg production on host worm burden [7].

We can characterize the resilience of the parasite population to periodic chemotherapy by analyzing its behavior at low worm burdens. At endemic worm burdens, worm acquisition among hosts is balanced by lower net egg output due to worm density in hosts. When worm burdens are reduced to low levels (for example, by treatment), there are no density-dependent effects and worm burden growth is at a maximum. In the presence of a program of regular treatment, we can define a net growth rate made up of the loss of worm burden to a round or treatment and its subsequent recover up to the next round of treatment. For the purposes of the model, the treatment program is defined by a series of treatments applied to a fraction, *g*, of school-age children using a drug with efficacy, *h* (the proportion of worms killed by one treatment in the treated host). Treatments are given repeatedly and are separated by an interval of *τ* years. Within the model, the individuals treated are assumed to be chosen at random, and hence the net treatment efficacy, *γ*, is given by the product of *g* and *h*. Non-compliance is not currently included in our model.

The details of the analysis are presented in Text S1, Section A. The result is a growth factor per treatment interval, *q*. That is, the worm burden of the population will increase by a factor *q* across a single round of chemotherapy and the parasite's response to the therapy up to the next treatment round. In this regard, *q* can be viewed as analogous to an effective reproduction number. If each worm produced a single generation of *q* new worms after an interval of 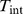 (the interval between rounds of treatment), the long term growth rate would match the current model. The exponential growth rate for the worm burden is given by 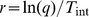. It is also possible to directly calculate the mean effective reproduction number of the parasite under a regular school-based treatment program. In the Text S1, Section A, we derive an expression for *R_e_* as
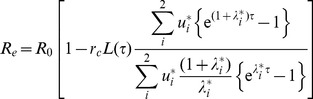
(4)where *R_0_* is basic reproduction number and the quantities λ*, u* and *L(τ)* are also defined in the SI. The term in brackets is the fractional impact on the reproduction number due to the treatment regime. The treatment regime will eradicate the parasite if *R_e_*<1. In Text S1, Section B and Figures S1 and S2, we compare these two measures of growth rate.

The model described by equations (1–2) ignores the effect of sexual reproduction and assumes that all eggs generated by female worms in the host population are fertile (non-sexual reproduction or non-SR model). In reality, the production of fertile eggs by female worms requires the presence of at least one mature male worm. Several models of the worm mating process have been proposed [9], [20]), but we focus on the polygamous model which assumes that the presence of a single male ensures that all eggs will be fertilized. It has the advantage of conceptual simplicity as well as allowing the mean fertile egg production rate to be calculated in a closed form. To include the effect of sexual reproduction, the egg production function 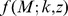 needs to be multiplied by the mating probability factor, *φ*, where
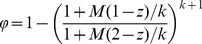
(5)


**Figure 1:**
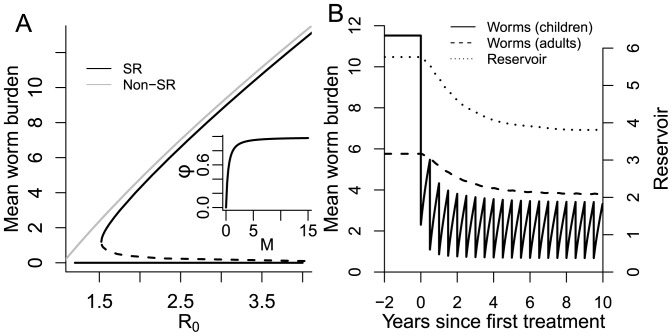
A) Solutions of equations (1–2), without age structure, both with and without mating probability factor φ. Broken lines represent unstable solutions [Inset: Mating probability factor φ as a function of mean worm burden]. B) Time series for mean worm burden in children and adults and reservoir content in response to annual treatment.

We will refer to the resulting model as the sexual reproduction or SR model. The factor 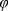 is effectively the fraction of total egg production from the host population that is fertilized. For large values of *M*, *φ* is effectively equal to 1 and sexual reproduction has no influence. For low values of *M*, *φ* approaches zero, indicating the decreasing probability of a female worm co-infecting with a male. As can be seen from Figure 1A (inset), there is no clear boundary for the effect of sexual reproduction, but it has a strong impact on fertile egg production for mean worm burdens of less than about 2.5. We define this approximate cut-off point as *M_SR_*. For worm burdens below *M_SR_*, the decline in fertile egg production reaches a point at which it balances the ability of the worms and infectious material to persist in the environment, defining a ‘breakpoint’ [9], [20], [21]). Below the breakpoint is a stable parasite-free state. The breakpoint is generally at very low values of mean worm burden and has a minimal effect on the normal endemic state of the parasite population, except at low values of *R_0_* at which the endemic solution disappears [9] (See Figure 1A, main panel).

The default parameter values used in simulations are given in Table 1. They represent a scenario for *A. lumbricoides* in a community where children have twice the exposure to eggs in the reservoir and also contribute twice as much to that reservoir by comparison with the remaining population age groups. Treatment is annual with an net efficacy of 80%, reflecting the high efficacy of a treatment like mebendazole (95%) and high school attendance levels of around 85%.

**Table 1:** Dimensional and non-dimensional model parameters with default values used in all calculations (unless otherwise stated).

Dimensional parameters		
Parameter	Value	Source
Aggregation parameter, k	0.7	[25], [26]
School-aged fraction of population, n_c_	0.3	[17]
Average egg survival time, 1/μ	0.2 yrs	[27]
Average worm lifespan, 1/σ	1 yrs	[28]
Relative contact rate of children, β_c_	2	See text
Relative contribution of children, p	2/3	See text
Treatment interval, τ	1 yr	See text
Treatment efficacy, gh	0.8	[17]
**Non-dimensional parameters**		
Effective treatment interval, 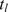	1	-
Basic reproduction number, *R_0_*	3	[29]
Fraction of R_0_ due to children, 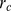	0.63	-
Net treatment efficacy, 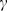	0.8	-
Relative reservoir timescale, 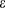	0.2	-

## Results

### Behaviour without sexual reproduction

We first examine the stability of the parasite dynamics in the non-SR model (equations 1–2) under annual treatment of school-age children in the absence the effect of sexual reproduction. Figure 1B shows the impact of school-age deworming on the three variables of the model – mean worm load in children, mean worm load in the remaining population, and the reservoir of infectious material in the environment. Treatment produces an immediate effect on the worm burden of children, but recovery is also very rapid, due to re-infection from material in the infectious reservoir. Reduced output of eggs from children allows the reservoir level to drop which in turn is reflected in worm burden in the adult portion of the population.

Analyses presented in the appendix (Text S1, Section A) show that, in the absence of sexual reproduction, the quantities *q* and *R_e_* can be expressed in terms of just five parameter groupings which capture the key epidemiological processes influencing the impact of mass treatment for STH infection (see SI):

- *R_0_*, the basic reproduction number for the parasite in the absence of effects induced by population density within the human host and the probability of being mated;
- *r_c_*, the fraction of *R_0_* attributable to school-age children, capturing the essence of the social structure of the population. It is defined as 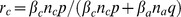;
- *t_l_* is the effective treatment interval, the length of the treatment interval as a fraction of the mean worm lifespan in the host and it captures the timescale of the treatment with respect to the dominant time scale influencing the parasite's population dynamics;
- *γ*, the net efficacy of the treatment in the targeted population which is the product of fraction, *g*, of school-age children using a drug with efficacy, *h*; and
- *ε*, viable life of eggs in the reservoir as a fraction of mean worm lifespan or the relative timescale of the reservoir of infectious material.

**Figure 2:**
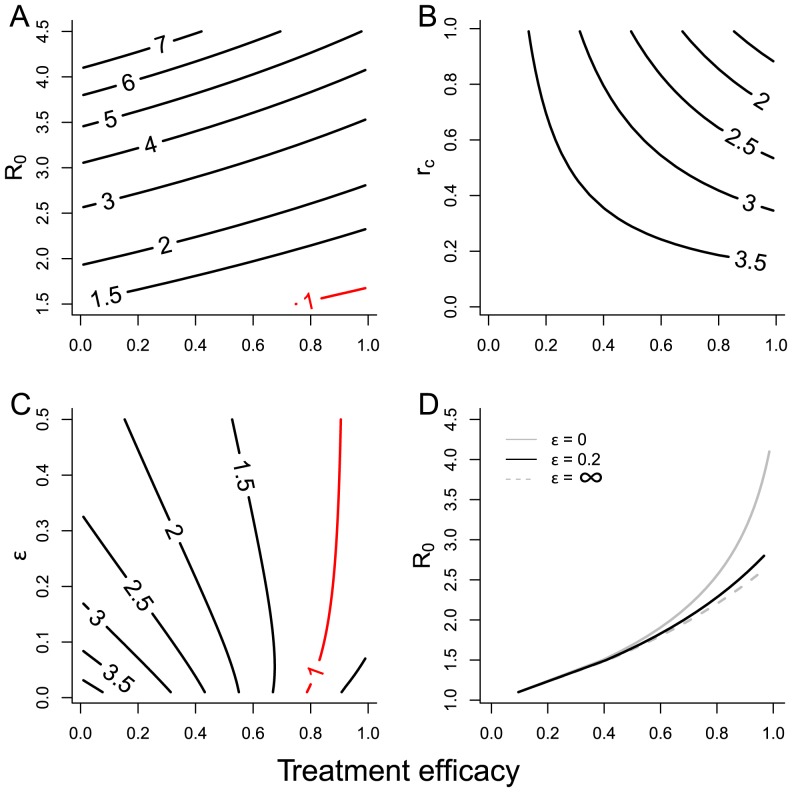
Dependence of growth rate, k, on A) R_0_ and effective treatment, γ; B) contribution of children to parasite reproduction, rc, and effective treatment; C) relative reservoir timescale, ε, and effective treatment. D) Extinction point for parasite for different values of ε. [For C, R_0_ = 2.5 and D, r_c_ = 1].

In Figure 2, we examine how the resilience of the model to treatment, as expressed by *q*, depends on these key parameters. Figures 2 A and 2B illustrate the impact of treatment efficacy on *R_e_* as a function of *R_0_* and *r_c_*, respectively. Figure 2A shows that the effect of treatment on *q* is approximately linear for the range of *R_0_* which we are considering. Fully effective treatment reduces q by about 40%. Extinction of the parasite is only possible for low *R_0_* (around 1.5) and the highest levels of effective treatment coverage (close to 100% with efficacy of 70%).

This reduction is strongly dependent on the relative contribution of children to the infection process, *r_c_*, as shown in Figure 2B. As *r_c_* increases and children play a more important role in transmission, the impact of targeted age-specific treatment on transmission also increases. However, even when children are solely responsible for transmission (*r_c_* = 1), the parasite is not wholly eradicated. This is due to the infectious reservoir, in which the parasite can persist in egg form, unaffected by chemotherapy. The dynamics of the reservoir are to a large extent determined by the effective lifespan of infectious material, which is very sensitive to environmental conditions [22]. Studies for hookworm suggest 3–4 week life expectancy under favorable conditions[23]. Reported life expectancies for *A. lumbricoides* eggs are significantly longer [22]. The influence of the infectious lifespan of material in the reservoir is illustrated in Figures 2C and 2D. The lifespan of the reservoir is captured solely by the parameter *ε*, which is the viable life of eggs in the reservoir as a fraction of mean worm lifespan. Figure 2C shows the resilience of the parasite as a function of ε and the effective fraction treated. To allow extinction to appear within the range of parameters scanned, *R_0_* is reduced to 2.5 and *r_c_* set to 1. For low treated fractions, a faster turn-over of the reservoir (smaller *ε*) leads to higher values of *q*. The stability of the parasite population is increased by having more worm lifecycles between treatment rounds. However, for parameter values close to the extinction contour (coloured red in the figure), a shorter lifespan for reservoir material leads to a parasite population that is less resilient to regular chemotherapy. The reservoir represents a source of new worms to repopulate the treated hosts. The longer the lifespan of reservoir material, the greater is its ability to re-infect after chemotherapy. The extent of this effect is limited, however. Figure 2D shows the critical combinations of *R_0_* and treatment for extinction of the parasite under different values of *ε*. The two grey lines mark out the extremes of behavior at very long lifespans for infectious material to very short. The latter matches the usual assumption of a reservoir that equilibrates much faster than the worm lifespan and is the usual assumption made in models [8], [15], [16]. For values of *R_0_* greater than 2, the difference between the two scenarios in the possibility of extinction is quite pronounced. We note also that the default value for *ε* = 0.2, indicating a reservoir timescale 5 times shorter than worm lifespan, is much closer to the slow reservoir assumption than the usual fast assumption.

### Behaviour with sexual reproduction

**Figure 3:**
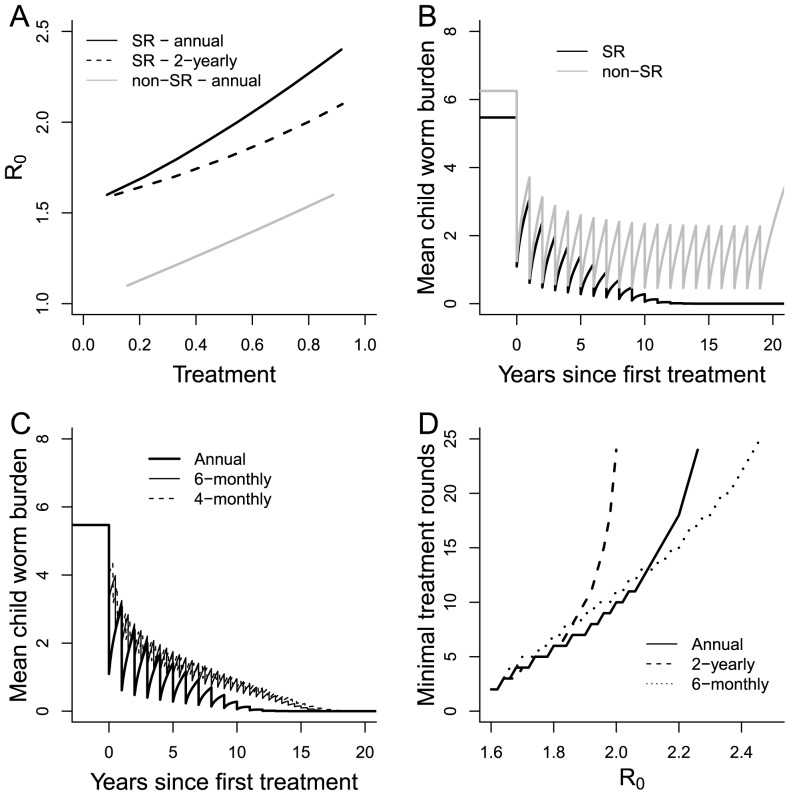
A) Critical treatment efficacy for SR and non-SR dynamics and different treatment intervals. B) Evolution of worm burden in children under annual treatment with and without sexual reproduction dynamics (default parameter values and R_0_ = 2) C) Time series showing effect of different intervention frequencies with same annual treatment rate. D) Minimum number of treatment rounds necessary to achieve elimination (with SR) as a function of R0 and the interval between treatments.

We now examine the effect of including the dynamics of sexual reproduction in the host into the model. A commonly made assumption is that the sexual reproduction mechanism has a negligible impact on parasite dynamics except at the lowest worm loads. This situation is illustrated by Figure 1A, which shows equilibrium worm burden as a function of *R_0_* with and without sexual reproduction. Significant discrepancies arise only for *R_0_* values around 1.5 and lower and result from the assumption implicit in standard *R_0_* calculations that female worms still generate fertile eggs at very low population levels. Figure 3A contrasts the critical treatment efficacies for models with (labelled SR) and without (labelled non-SR) sexual reproduction as a function of *R_0_*. It is clear that, in general, the presence of the sexual reproduction mechanism in the model makes interrupting transmission much easier, placing it now at the low end of measured *R_0_* values (1.5–2.5) for an annual treatment regime. Even for 2-yearly intervention, elimination is possible for *R_0_*<2.

The effect of the introduction of SR can be understood by looking at the form of the mating probability factor, *φ* (See Figure 1A and equation 5). The value of *φ* drops significantly below 1 only when the mean worm burden is less than about 2. Therefore it is only when worm burdens drop below this level that SR starts to have a limiting effect on net parasite transmission within a community. Figure 3B illustrates this effect. It shows, under annual treatment, changes over time in the mean worm burden among school-age children, both with and without sexual reproduction, for the default parameter values but with *R_0_* = 2, to better illustrate conditions under which elimination is possible. Initial rounds of treatment have very similar impacts on the mean worm burden of in both the SR and non-SR context. However, in the case of SR, the effect of mate scarcity for worms in children means that the recovery rate is slightly reduced with respect to the non-SR regime. Over a number of treatment rounds, the effect of impaired recovery of the parasite population accumulates until it can no longer support itself and is eliminated. For the case shown in Figure 3B, the worm population is sufficiently reduced after 10 rounds of annual treatment that it will not recover even if regular treatment is stopped. This non-linearity in worm recovery at low burdens has consequences for how the capacity for treatment is distributed over time. Figure 3C shows the same annual coverage (80%) applied annually, twice a year (at 40%) or three times a year (at 26.7%). For the latter two cases, the effect of treatment of treatment is identical in the long term. Individual rounds of treatment do not force the worm burden of children down to the SR-limiting levels. Parasite recovery is linear and only the mean rate of treatment is important. However, when treatment is concentrated in a single annual intervention, recovery is impaired by the low levels temporarily achieved, resulting in elimination of the parasite in 10 rather than 15 years.

Figure 3D further illustrates this phenomenon. It shows the minimum number of treatment rounds necessary to achieve elimination as a function of *R_0_* and the interval between treatments. It is clear that to eliminate the parasite with a given treatment interval requires more rounds of treatment as *R_0_* increases. For higher values of *R_0_*, fewer rounds of treatment, delivered at a higher frequency, can eliminate the parasite. Comparing treatment intervals for *R_0_* of 2 or less, there is little dependence on treatment frequency as opposed to absolute number of treatment. More surprisingly, comparing annual and 6-monthly frequencies, the higher frequency requires more rounds of treatment to reach elimination. This behavior can be seen as a facet of the time-scale of the parasite infection cycle. Only worms in children are amenable to chemotherapy. Those in adults or (effectively) in the reservoir are protected from it. By treating too frequently, we simply retreat already treated individuals. Treating more slowly, we allow worms in other parts of the system to come ‘within range’ of school-based chemotherapy and hence we use the treatment more efficiently.

## Discussion

Analysis of the dynamics at low worm burdens showed that the response of the model to long-term periodic treatment is governed by the five parameter groupings mentioned in the methods section; *R_0_*, *r_c_*, *t_l_*, *γ* and *ε*. Briefly, *t_l_* and *γ* are properties of the intervention strategy and the efficacy of the drug used. *R_0_*, the basic reproduction number, is a ‘summary’ parameter for intensity of transmission, which includes the natural history of the parasite within the host and its interaction with the hosts' environment. A range of estimates for *R_0_* have been calculated for different species by a variety of methods [9], but there is a need for better and more estimates in settings of low, medium, and high transmission.

The parameter grouping *r_c_* has not been defined before. It represents an amalgamation of the relative rate of contamination from the different age groups and their relative exposure to the material in the environment. It is large if SAC play a dominant role in the transmission cycle for the parasite and low if it is dominated by adult contributions. These analyses suggests that the impact of an intervention can be very sensitive to its value. If children are largely responsible for contaminating the home and surrounding environments, then the impact of school-age treatment is enhanced with respect to the effective reproductive number and mean worm loads in the rest of the community. It is the macroparasitic equivalent to the ‘Who Aquires Infection From Whom’ (WAIF) matrix central to models for flu and childhood infectious diseases (see [9]). At present, understanding of such non-random exposure to infective stages by different age groups is very limited. While relative exposure of age groups can be inferred from infection age profiles [19], [24], estimating the relative contributions of age groups to the contamination of the environment will require careful examination of post-treatment infection dynamics in different age classes.

A second important aspect of this work is the significance of the lifetime of infectious material in the environment as represented by the parameter grouping, *ε*. Most previous models assume that dynamics of the infectious reservoir are fast and can be assumed to be in equilibrium as parasite dynamics are concerned. In common with the influence of SR on parasite populations, this assumption is well founded in the absence of regular interventions. Under periodic treatment of a section of the host population, however, the reservoir is a source of new infection beyond the reach of treatment and the longer the lifespan of material, the more ‘infectious potential’ it has. Figure 2D shows that reservoir dynamics can have significant effect on the resilience of the parasite in the host in the absence of SR, with a longer lifespan increasing the level of treatment necessary to achieve elimination. When SR is included, the sensitivity of the parasite to reservoir dynamics increases markedly. Currently, little is known about how infectious material is transmitted between hosts and what its dynamics are in the environment. This study suggests that a better understanding of these issues could help considerably in constructing accurate models and designing control programs. Furthermore, seasonal treatment timed to match a period of dry hot weather when infectious material lifespans are shorter may also enhance the impact of mass drug administration.

The dioecious nature of STHs require females to find a mate within their human host to produce fertile infective stages. The difficulty in doing this is, to a large extent, governed by the frequency distribution of parasite numbers per human host. For intestinal worms, endemic mean burdens tend to be low (e.g. 10–20 for *A. lumbricoides*, [9], [25]), but still significantly greater than the levels at which finding a mate becomes a problem, as defined by the worm burden value *M_SR_* at which finding a mate becomes problematic which is typically around a mean of one worm per host.

Mass treatment interventions have the effect of substantially reducing worm burdens in a large section of the population, such as school-age children. As a result, the burden in a particular population group can easily drop below *M_SR_*, if only temporarily, and mating dynamics can start to have a negative impact on parasite reproduction and subsequent transmission potential as there are fewer and fewer instances of both sexes infecting the same individual. Hence, it is clear that in attempting to define optimum repeated mass treatment programs that aim to reduce mean worm burden within a population to a level of *M_SR_* or below, due note must be taken of the mating factor. In the context of the current work, this would be a goal of approximately a 70% reduction in worm burden in a given age category. As discussed in the results section, the breakpoint, below which elimination is assured, can be achieved cumulatively. Figure 3D indicates that for *R_0_* of 2 and treatment efficacy of 80%, this point is reached after about 10 rounds of annual treatment. The effects noted here for the elimination of STH may also apply to other sexually-reproducing parasitic worm infections such as schistosomiasis. However, given the much greater numbers of worms per host for schistosomes, these effects may be insignificant.

A clear message from our work is that in order to analyze the dynamics of the parasite population near or at the point of elimination, sexual reproduction dynamics are an essential component of a model. This includes attempts to design long-term programs leading to elimination as well as the expected consequences of the failure of such programs or subsequent reintroductions after elimination. The interaction of the non-linearity of sexual reproduction with the highly temporally localised effect of mass drug administration can lead to some unusual effects which have potential consequences for the design of future control policies, particular those targeted towards parasite elimination. Figure 3C shows that the effect of a given level of drug delivery given in one annual delivery is significantly different from the quantity of drugs spread over two or three proportionately smaller deliveries. A single delivery, if the coverage is large enough, precipitates mate scarcity in the treated group and hence slows recovery as compared to the same coverage over several treatments. As a result, elimination can be achieved substantially sooner. Note also that the effect of 40% coverage twice yearly and 27% coverage three times a year are effectively identical, since for these smaller treatment impacts the model's response is effectively linear. This is basically the assumption of a previous method of estimating the impact of periodic treatment on worm burden [9]. From our analysis of the effect of sexual reproduction on worm burden recovery, this approach will clearly lead to an underestimate of impact when coverage and efficacy are high. Assuming that the number of rounds of treatment is probably the major component determining the cost of an elimination program, Figure 3D indicates that, for low *R_0_*, the cost of a successful program may be largely independent of intervals between treatments. Indeed, the fact that 6-monthly treatment requires more rounds than annual suggests that there may be an economically optimal frequency of treatment for a given parasite and treatment strategy in a defined transmission setting. Future work will investigate this issue.

In conclusion, the analyses point to the need for better designed field studies to measure the parameter combinations defined by the models, if the design of MDA programs is to be improved. Models play a key role in defining what to measure if a better understanding of the effect of treatment on the parasites transmission dynamics is to be achieved.

## Supporting Information

Figure S1 **Dependence of q (Panel A) and Re (Panel B) on R0 and the effective fraction treated.**
(EPS)

Figure S2 **Dependence of q (Panel A) and Re (Panel B) on the timescale parameter ε and the effective fraction treated.**
(EPS)

Text S1 **Section A – Calculating the growth rate of the parasite population under regular treatment; Section B – Comparing the largest eigenvalue** ***q*** **with** ***R_e_*** **.**
(DOCX)
