# Supplementary material for: Modeling the Interruption of the Transmission of Soil-Transmitted Helminths by Repeated Mass Chemotherapy of School-Age Children
Source: PLoS Negl Trop Dis. 2014 Dec 4;8(12):e3323. doi: 10.1371/journal.pntd.0003323 (PMC4256169; doi:10.1371/journal.pntd.0003323)
Supplement: Text S1 — Section A – Calculating the growth rate of the parasite population under regular treatment; Section B – Comparing the largest eigenvalue q with Re . (DOCX) [file pntd.0003323.s003.docx]

**The interruption of the transmission of soil transmitted helminths by repeated mass chemotherapy targeted at school aged children**

James Truscott^1^, T. Déirdre Hollingsworth^234^ and Roy Anderson^1^

^1^London Centre for Neglected Tropical Disease Research, Department of Infectious Disease Epidemiology, Faculty of Medicine, Imperial College London, St Marys Campus, Norfolk Place, London W2 1PG

^2^Mathematics Institute, University of Warwick, Coventry, CV4 7AL, UK

^3^School of Life Sciences, University of Warwick, Coventry, CV4 7AL, UK

^4^Department of Clinical Sciences, Liverpool School of Tropical Medicine, Pembroke Place, Liverpool, L3 5QA, UK

# **Supporting Information**

# A: Calculating the growth rate of the parasite population under regular treatment

The application of the concept of reproduction number to STHs is a little problematic. The mechanism of sexual reproduction generates non-linear behaviour for the very low worm burdens at the very threshold where linear behaviour is necessary for the definition of classical reproduction numbers (the ‘breakpoint’). In this first analysis, we neglect the issue of sexual reproduction and assume that all eggs produced by female worms are fertilised. The effects of this assumption are analysed in more detail in the main text.

The equations governing the two-component plus reservoir system are

$$\begin{matrix} \frac{dM_{c}}{dt}=\beta_{c}l-\sigma M_{c}, \\ \frac{dM_{a}}{dt}=\beta_{a}l-\sigma M_{a}, \\ \frac{d}{dt}l=\frac{R_{0}\mu\sigma}{\lambda z\left(\beta_{c}n_{c}p+\beta_{a}n_{a}q\right)}\left[f\left(M_{c};k,z\right)n_{c}p+f\left(M_{a};k,z\right)(1-n_{c})(1-p)\right]-\mu l \end{matrix}$$

Where *M_c_* and *M_a_* are the mean worm burden in children and adults respectively and *l* is the level of infection in the environment. Further details are given in the main text.

Linearised around zero worm burden in both adults and children and scaling time by worm lifespan σ, we get

$$\frac{d}{dt}\left(\begin{matrix} M_{c} \\ M_{a} \\ l \end{matrix}\right)=\left(\begin{matrix} -1 & 0 & \beta_{c} \\ 0 & -1 & \beta_{a} \\ R_{0}A/\varepsilon D & R_{0}B/\varepsilon D & -1/\varepsilon\end{matrix}\right)\left(\begin{matrix} M_{c} \\ M_{a} \\ l \end{matrix}\right)$$

where $\varepsilon=\sigma/\mu$. The contact rates β_c_ and β_a_ are scaled by σ and$A=n_{c}p$, $B=n_{a}(1-p)$and $D=\beta_{c}A+\beta_{a}B$. The eigen-value/eigen-vector pairs for this system are $\lambda_{i},\mathbf{v}_{i}$, where $\lambda_{1}>\lambda_{2}>\lambda_{3}$. The eigen-values and vectors are given by

$$\begin{matrix} \lambda_{1}=\frac{-(1+\varepsilon)+\sqrt{(1+\varepsilon)^{2}+4\varepsilon(R_{0}-1)}}{2\varepsilon} \\ \lambda_{2}=-1 \\ \lambda_{3}=-(1+1/\varepsilon)-\lambda_{1} \end{matrix}$$

and

$$\mathbf{v}_{1}=\left(\begin{matrix} \beta_{c} \\ \beta_{a} \\ \lambda_{1}+1 \end{matrix}\right),\mathbf{v}_{2}=\left(\begin{matrix} B \\ -A \\ 0 \end{matrix}\right),\mathbf{v}_{3}=\left(\begin{matrix} \beta_{c} \\ \beta_{a} \\ \lambda_{3}+1 \end{matrix}\right)$$

The evolution of the system can be described by

$$\left(\begin{matrix} M_{c}(t) \\ M_{a}(t) \\ l(t) \end{matrix}\right)=\mathbf{X}(t)=\sum_{i} w_{i}e^{\lambda_{i}t}\mathbf{v}_{i}$$

We now let,

$$\mathbf{M}=\left(\begin{matrix} \mathbf{v}_{1}, & \mathbf{v}_{2}, & \mathbf{v}_{3} \end{matrix}\right)$$

Then M transforms coordinates in the eigen-vector space into real space (*M_c_*, *M_a_* and *l*) and M^-1^ back the other way. To describe the dynamics of the periodic treatment regime, we need two other operators. Firstly a treatment operator, T;

$$\mathbf{T}=\text{diag}(1-\gamma,1,1)$$

which acts on real state vector and describes the reduction of children’s worm burden by $1-\gamma$. Also a growth operator,

$$\mathbf{G}=\text{diag}(\exp(\lambda_{1}\tau),\exp(-\tau),\exp(\lambda_{3}\tau))$$

which acts on the eigen-state vector and gives the state after a period of growth of length τ. So the sequence of a complete cycle of treatment is

$$\mathbf{\omega}_{n+1}=\mathbf{M}^{-1}\mathbf{TMG}(t_{l})\mathbf{\omega}_{n}=\mathbf{H\omega}_{n}$$

where **ω** is the state of the system in eigen-state space. The parameter tl is the interval between treatment, scaled by worm lifespan, σ. Hence the dynamics of the system is dependent on the largest eigen-value/vector pair of the mapping matrix **H**.

$$\mathbf{Hu}=q\mathbf{u}$$

When $k\leq1$, the parasite will be eliminated from the system. The matrix, H, is given by

$$\mathbf{H}=\left(\begin{matrix} 1-r_{c}\gamma\varsigma& -\frac{Br_{c}\gamma\varsigma}{\beta_{c}} & -r_{c}\gamma\varsigma\\ -\frac{\gamma\beta_{a}\beta_{c}}{D} & 1-(1-r_{c})\gamma& -\frac{\gamma\beta_{a}\beta_{c}}{D} \\ -r_{c}\gamma(1-\varsigma) & -\frac{Br_{c}\gamma(1-\varsigma)}{\beta_{c}} & 1-r_{c}\gamma(1-\varsigma) \end{matrix}\right)\mathbf{G}(\tau)$$

where $r_{c}=\beta_{c}A/D$ and $\varsigma=-(1+\lambda_{3})/(\lambda_{1}-\lambda_{3})$. The eigenvector q tells us the growth in the parasite population over a single treatment interval. One interpretation of *q* is as a crude effective reproduction number, with each worm generating q off-spring worms after an interval of τ years. In terms of an equivalent continuous growth rate, $r=\ln(q)/t_{l}$ or in dimensional units

$$r=\frac{\ln(q)}{T_{\mathrm{int}}}$$

At a time $0\leq t\leq\tau$, the state of the system in real coordinates is proportional to $\mathbf{MG}(t)\mathbf{u}$. Hence

$$M_{n}(t)=\sum_{i,j}^{3} M_{ni}G_{ij}(t)u_{j}=\sum_{i}^{3} M_{ni}\exp(\lambda_{i}t)u_{i},$$

where n=1 for children and 2 for adults.

# Calculating the effective reproduction number, *R_e_*

If we know the number of eggs generated by worms in both types of hosts and the relative worm populations within each type of host, we can calculate the total number of new worms generated and hence the effective reproduction number. Under treatment, the number of eggs generated by a worm will depend on when it is ‘born’ in the treatment cycle (between 0 and t_l_). Egg production is assumed constant with time but the probability of a worm surviving treatment for time t is$\exp(-t)$, with a factor of 1-γ included for each treatment round. The mean life of a worm is the time-integral of the survival function,

$$\bar{S}_{c}(t_{0})=1-\frac{\gamma\exp(-t_{l})\exp(t_{0})}{[1-(1-\gamma)e^{-t_{l}}]}=1-L(t_{l})\exp(t_{0})$$

where $0\leq t_{0}\leq t_{l}$is the time of birth of the worm in the treatment cycle. For worms in adults, γ=0, and $\bar{S}_{a}(t_{0})=1$. The output from each worm can then develop into new worms of either type. Hence we can define an effective R for a worm born at time $t_{0}$in either a child or adult

$$\mathbf{R}(t_{0})=\left(\begin{matrix} \frac{R_{0}A(\beta_{c}+\beta_{a})}{D}(1-L(t_{l})\exp(t_{0})) \\ \frac{R_{0}B(\beta_{c}+\beta_{a})}{D} \end{matrix}\right)=\left(\begin{matrix} \frac{R_{0}r_{c}(\beta_{c}+\beta_{a})}{\beta_{c}}(1-L(t_{l})\exp(t_{0})) \\ \frac{R_{0}(1-r_{c})(\beta_{c}+\beta_{a})}{\beta_{a}} \end{matrix}\right)$$

The number of worms born at any time is the incidence, $I(t)$, and depends on the time within the treatment cycle.

$$\frac{dM_{k}}{dt}=I_{k}-M_{k}$$

where *k* is either *c* or *a* (child or adult). So

$$I_{k}(t)=e^{-t}\frac{d}{dt}\left\{e^{t}M_{k}(t)\right\}$$

Substituting the expression for the evolution of the worm burdens gives

$$I_{k}(t)=\sum_{i}^{3} M_{ki}u_{i}(1+\lambda_{i})\exp(\lambda_{i}t)=\sum_{i=1,3} M_{ki}u_{i}(1+\lambda_{i})\exp(\lambda_{i}t)$$

for host type *k*. Note that the i=2 term doesn’t contribute since $\lambda_{2}=-1$. As a result, the above expression for *I_k_*(*t*) can be written in terms of vectors and matrices of degree two. That is

$$I_{k}(t)=\sum_{i}^{2} M_{ki}^{*}u_{i}^{*}(1+\lambda_{i}^{*})\exp(\lambda_{i}^{*}t)$$

where M* is made up of the first and second row and first and third columns of M and u* comprises the first and third elements of u. The vector of eigen-values is now made up of $\mathbf{\lambda}^{*}=\left(\lambda_{1},\lambda_{3}\right)$.

The overall reproductive number, R_e_, is the average R over a treatment cycle, weighted by the incidence of new worms in each type of host at any given time.

$$R_{e}=\frac{\int_{s=0}^{t_{l}} \sum_{j}^{2} R_{j}(s)I_{j}(s)ds}{\int_{s=0}^{t_{l}} \sum_{k}^{2} I_{k}(s)ds}$$

This expression can be considerably simplified. The definition of ***R***(t) can be split into a constant and a time-dependent term,

$$\mathbf{R}(t_{0})=R_{0}(\beta_{c}+\beta_{a})\left\{\left(\begin{matrix} r_{c}/\beta_{c} \\ (1-r_{c})/\beta_{a} \end{matrix}\right)-\left(\begin{matrix} r_{c}L(t_{l})/\beta_{c} \\ 0 \end{matrix}\right)\right\}\exp(t_{0})=\mathbf{R}^{a}-\mathbf{R}^{b}(t_{0})$$

Note also that from the definition of **M** above, $M_{ij}^{*}=\beta_{i}$. As a result, effective R can be expressed

$$R_{e}=R_{0}\left[1-r_{c}L(t_{l})\frac{\sum_{i}^{2} u_{i}^{*}\left\{\mathrm{e}^{(1+\lambda_{i}^{*})t_{l}}-1\right\}}{\sum_{i}^{2} u_{i}^{*}\frac{(1+\lambda_{i}^{*})}{\lambda_{i}^{*}}\left\{\mathrm{e}^{\lambda_{i}^{*}t_{l}}-1\right\}}\right]$$

Finally, we need to calculate **u***, which comprises the first and third elements of **u** defined by $\mathbf{Hu}=q\mathbf{u}$. By scaling **u**, the simplest form for u* is

$\mathbf{u}*=\left(\begin{matrix} \varsigma(e^{\lambda_{2}^{*}t_{l}}-q) \\ (1-\varsigma)(e^{\lambda_{1}^{*}t_{l}}-q) \end{matrix}\right)$.

The key stability indicators for this system are therefore expressible purely in terms of 5 non-dimensional parameters; *R_0_*, *γ*, *r_c_*, *t_l_* and *ε*.

## B: Comparing largest eigenvector q with *R_e_*

In Section A, above, we calculated two complementary measures for the stability of the parasite population in the absence of sexual reproduction. The effective reproduction number, *R_e_*, captures the ability of the worms to generate new adults within their reproductive lifetime while being subject to regular treatment. It captures the essential natural history of the parasite but no information about time scale of its constituent stages. The parameter q represents the growth of the parasite population as a whole in the interval between treatments. It captures the bulk behaviour of the parasite population and the timescale on which the changes happen. As shown in Figure 1, *R_e_* and q match qualitatively for variation in parameters that don’t affect relative timescales of parasite development, such as *R_0_*, level of treatment (γ) and relative contribution of children (r_c_). However, for the parameter ε, that governs the relative lifespans of the adult and infectious phase of the parasite, there are significant differences. Figure 2A and B compare the values q and *R_e_* against ε and treatment level. Both measures pick up stabilising effect of a longer reservoir timescale (larger ε). This is a consequence of the changing distribution of infectious material among the different forms in the parasite lifecycle and is a feature of the natural history of the parasite. However, only q picks up on the increasing growth-rate for short reservoir timescales (lower ε) at lower levels of treatment. Thus the parameter q picks up the increased resilience in the parasite population; what might be thought of as the faster overall bounce-back time of the population under regular treatment. This information is absent from *R_e_* since it is purely a timescale phenomenon.

Figure Captions:

Figure 1: Dependence of q (Panel A) and Re (Panel B) on R0 and the effective fraction treated.

Figure 2: Dependence of q (Panel A) and Re (Panel B) on the timescale parameter ε and the effective fraction treated.
